# Predictable chronic mild stress promotes recovery from LPS-induced depression

**DOI:** 10.1186/s13041-019-0463-2

**Published:** 2019-05-03

**Authors:** Ruili Dang, Yan-yan Guo, Kun Zhang, Pei Jiang, Ming-gao Zhao

**Affiliations:** 1 Department of Pharmacy, Precision Pharmacy & Drug Development Center, Tangdu Hospital, Fourth Military Medical University, Xi’an, 710038 Shaanxi China; 2 Institute of Clinical Pharmacy & Pharmacology, Jining First People’s Hospital, Jining Medical University, Jining, 272000 Shandong China; 3 Department of Pharmacology, School of Pharmacy, Fourth Military Medical University, Xi’an, 710032 Shaanxi China

**Keywords:** Predictable chronic mild stress, Neuroinflammation, Depression, NLRP3

## Abstract

Clinical depression is frequently comorbid with chronic inflammatory disease, and neuroinflammation is currently proposed as a key mechanism in major depressive disorders. Different from unpredictable chronic stress, which is a well-established animal model for depression, predictable chronic mild stress (PCMS), a routine stress experienced in day-to-day life, has been demonstrated to improve mood and memory. In the present study, we assess the effects of PCMS (5 min of daily restrain stress for 4 weeks) on depressive-like behavior, neuroinflammation, oxidative stress, and pyrin domain containing three (NLRP3) activation in hippocampus of mice subjected to peripheral immune challenge by lipopolysaccharide (LPS). We found that PCMS facilitated the recovery from LPS-induced depressive- or anxiety-like behavior. Concurrent with the reversal of abnormal behavioral changes, PCMS suppressed LPS-induced proinflammatory cytokine expression, microglia activation, and oxidative stress in hippocampus. Correspondingly, PCMS inhibited LPS-induced overactivation of NLRP3 inflammasome components (NLRP3, ASC, and Caspase-1), and interleukin 1 beta (IL-1β) maturation. Nrf2 (nuclear factor (erythroid-derived 2)-like 2) signaling was demonstrated to inhibit NLRP3 inflammasome overactivation and oxidative stress. PCMS activated Nrf2 signaling and inhibited thioredoxin (Trx)-interacting protein (TXNIP) expression in LPS-treated mice. Collectively, present data suggest that PCMS, contrary to severe and uncontrolled stress, alleviated impairments of the Nrf2-TXNIP-Trx system and may contribute to inflammatory brain damage and the imbalance of cellular redox homeostasis in depressed mice. This study provides a mechanistic link to the resilience of PCMS to LPS-induced behavioral deficits.

**Electronic supplementary material:**

The online version of this article (10.1186/s13041-019-0463-2) contains supplementary material, which is available to authorized users.

## Introduction

The mechanisms responsible for major depressive disorder (MDD) are complex and not fully understood; however, exposure to chronic uncontrollable stress is widely believed to play a critical role in developing MDD [1, 2]. With increasing competitive pressure in modern society, individuals suffering MDD are rapidly increasing in number [3]. Thus far, a vast continuum of individual differences in coping with stress has been observed, and only a fraction of individuals exposed to stressful life events develop to psychiatric disorders, such as MDD and anxiety. Chronic unpredictable mild stress (CUMS) is a well-established procedure to induce depressive- and anxiety-like behavior in rodents [4, 5]. Acute stress, on the contrary, has been demonstrated to enhance adult rat hippocampal neurogenesis and improve memory performance [6]. Intriguingly, biphasic effects of stress are also shown in predictable chronic mild stress (PCMS) model (5 min of daily restraint stress for 4 weeks), where enhanced mood, learning, and memory are observed [7]. Thus, the overall effects of stress can be highly context-dependent and vary with the intensity, duration, or predictability. Severe and uncontrollable stressors are detrimental; however, tolerable stress with mild intensity and appropriate duration can promote health.

Beyond the central nervous system (CNS), the immune system, which consists of biological structures and processes, is also involved in the complex regulatory network of stress responses [7]. Findings over the past decade have implicated neuroinflammation in the etiology and maintenance of MDD [8]. Accumulating evidence has demonstrated that psychological or physical stressors can activate inflammation processes and production of inflammatory cytokines, leading to structural and functional alterations of neurons [9, 10]. Proinflammatory cytokines can in turn elicit profound behavioral changes that overlap with common symptoms of MDD. For example, inflammation-associated depression is often studied in rodents by peripheral administration of immunostimulants such as lipopolysaccharide (LPS) [11]. Systemic administration of LPS activated glial cells, expression of proinflammatory cytokines in the brain, and the occurrence of sickness, a behavioral state characterized by symptoms such as decreased motor activity and appetite, social withdrawal, and increased sensitivity to pain. These sickness responses are followed by a phase of depressive-like behavior that can be improved by antidepressant treatment [12]. Interleukin 1 beta (IL-1β) is believed as a critical mediator of depressive-like behavior caused by stress [14]. The Nod-like receptor family pyrin domain containing 3 (NLRP3) inflammasome is the major regulator of IL-1β maturation and release. Upon detection of various stress factors, assembly of the inflammasome protein complex results in activation and secretion of proinflammatory cytokines [13]. Activated NLRP3 has been found in MDD patients and animal models of depression [14]. Furthermore, NLRP3 has recently been proposed as a bridge that links the gap between immune activation with stress exposure, and potential pathogenic mechanism in the development of depressive symptomatology.

Inflammation is commonly associated with oxidative stress and the brain is highly sensitive to changes in redox status. Oxidative damage, including lipid peroxidation and DNA oxidation, is believed to play an important role in the pathogenesis of neuropsychiatric diseases [15]. Nrf2 (nuclear factor (erythroid-derived 2)-like 2) is a transcription factor that controls gene expression of cytoprotective proteins and enzymes, and comprises the most important cellular redox buffers. Growing evidence shows that Nrf2 inhibits inflammasome activation and consequently suppresses inflammation [16, 17]. More importantly, activating Nrf2 restores redox homeostasis and reverses vulnerability to depression after an intense stress [18]. Recent evidence has demonstrated that the thioredoxin (Trx) system, a key antioxidant system in the defense against oxidative stress, plays a critical role in activating the NLRP3 inflammasome. Under unstressed conditions, Trx1 is bound with Trx-interacting protein (TXNIP), and the NLRP3 remains inactive due to lack of TXNIP interaction with NLRP3. The dissociation of Trx1/TXNIP complex under oxidative stress, however, increases the TXNIP/NLRP3 interaction and activates the NLRP3 inflammasome [19]. Studies have suggested that Nrf2 inhibits NLRP3 inflammasome activation via regulating Trx1/TXNIP complex [16, 20], thereby making Nrf2-TXNIP-Trx signaling a potential mechanism that links neuroinflammation and oxidative stress to MDD susceptibility.

Based on the protective phenotype presented in the PCMS model, we assumed that repeated tolerable stress could increase adaptive capacity and enhance resiliency to negative effects of future stressors. Nrf2 signaling and inflammasomes represent stress response pathways that are considered as an attempt of affected tissues to cope with stressors and to restore a new homeostatic state. Thus, neuroinflammation and oxidative stress would be central to the vulnerability to depression. To this end, we examined the effects of PCMS on behavioral changes, NLRP3 inflammasome activation and Nrf2-TXNIP-Trx signaling on a background of inflammation. We found that PCMS facilitated recovery from LPS-induced depressive-like state and inhibited overactivation of NLRP3 inflammasome. PCMS also alleviated impairments of Nrf2-TXNIP-Trx system, providing a strong adaptive capacity that contributes to the resilience of PCMS to LPS-induced behavioral deficits.

## Materials and methods

### Animals

Eight-week-old C57BL/6 male mice were obtained from the Laboratory Animal Center of the Fourth Military Medical University. Mice were group-housed in a temperature-controlled (24 ± 2 °C) environment under constant 12/12 h light/dark cycle with free access to food and water. All animal procedures were carried out in accordance with the Regulations of Experimental Animal Administration issued by the State Committee of Science and Technology of the People’s Republic of China, with the approval of the Ethics Committee in Fourth Military Medical University.

### Experimental design

**Fig. 1:**
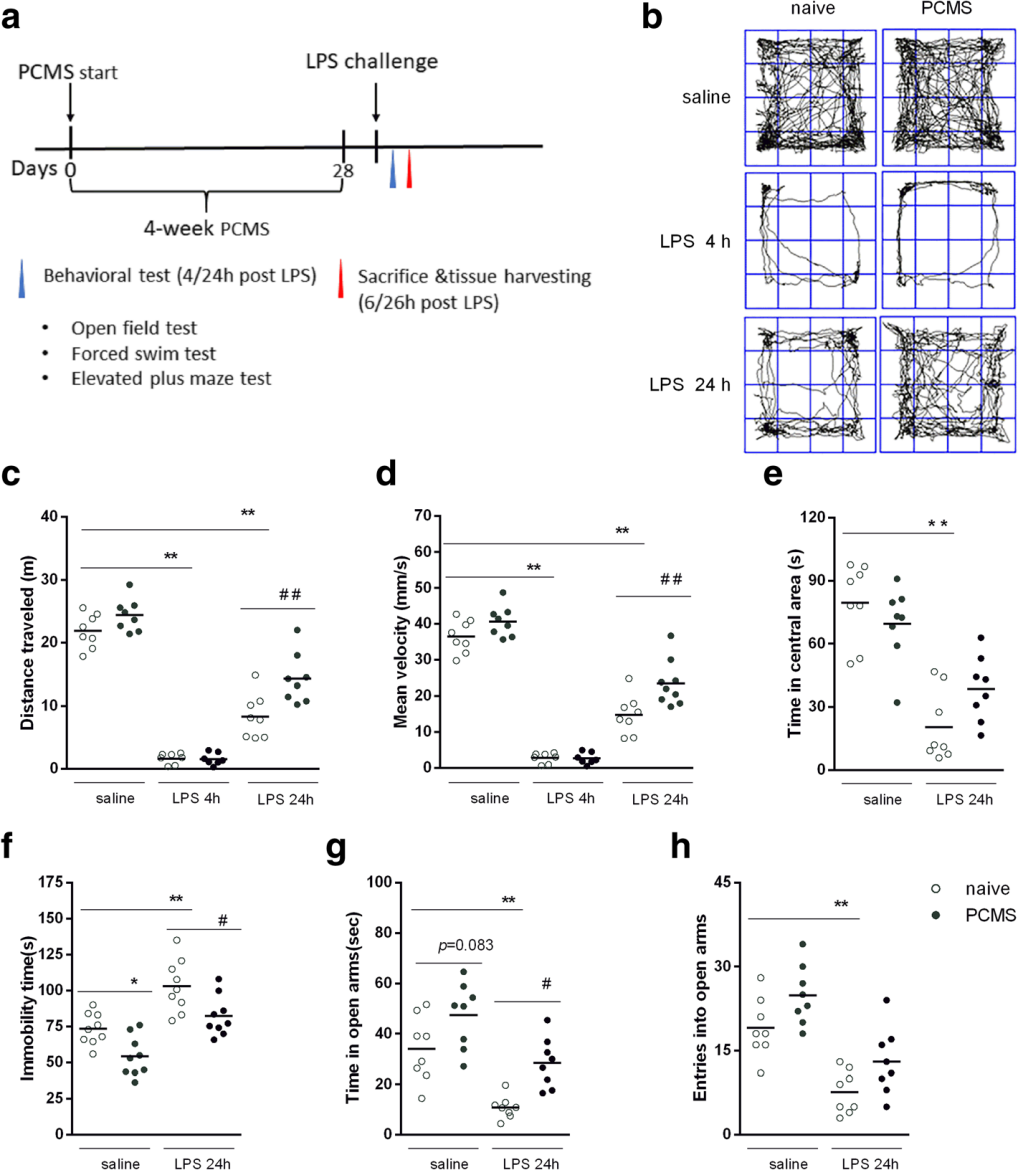
PCMS promotes recovery from LPS-induced depressive and anxiety-like behavior. Timeline of the experimental procedures (**a**). Sample traces of locomotor activity in the open field test (OFT) of naïve and PCMS mice 4 h and 24 h after saline or LPS (500 μg/kg) treatment (**b**). Total distance traveled (**c**), mean velocity (**d**) of locomotor activity and time spent in the center area (**e**) in the OFT. Immobile time in the forced swim test 24 h after saline or LPS (**f**). Time spent in the open arms (**g**), and entrance to open arms (**h**) in the elevated plus maze test. (*n* = 7–9). *p < 0.05, **p < 0.01 compared with the saline-treated naive group. ^#^*p* < 0.05, ^##^*p* < 0.01 compared with the corresponding LPS group

The PCMS protocol was conducted according to previous studies. Mice were initially guided to enter Plexiglas restrainer that had a ventilation grille on the head side and sliding door on the tail side and allowed to stay for 5 min with no mobility. At the end of 5 min, the mice were withdrawn gently from the restrainer and placed back into its home cage. To deduce the effects of handling on behavior, animals assigned to stress-naïve group underwent similar daily handling except the restraint stress procedure. The stress paradigm was conducted for four weeks at the same time each day (between 3:00 PM and 5:00 PM) to ensure that the time of the stress was predictable. On the next day after the last restraint session, LPS (200 or 500 μg/kg, *Escherichia coli* 055: B5, Sigma) or saline was administered via intraperitoneal injection. Sickness behavior is usually observed 3 h to 6 h following systemic LPS administration, whereas depressive-like behavior is observed 24 h after LPS challenge. Behavioral tests were thereafter conducted 4 h or 24 h after LPS injection. Immediately after the behavioral tests, mice were anesthetized with 10% chloral hydrate (4 mL/kg). Brain was carefully removed and immediately placed on ice. Hippocampus was removed with a micro-scissor and frozen in liquid nitrogen, and stored at − 80 °C until further analysis. The timeline of the experimental procedures is presented in Fig. 1.

### Behavior analyses

#### Open field test

The open field test (OFT) was carried out in a square arena (40 cm × 40 cm × 40 cm) with clear Plexiglas walls and floor, and placed inside an isolation chamber with dim illumination. Mice were placed in the center of the box and allowed to freely explore the arena undisturbed for 10 min. Mice were videotaped using a camera fixed above the floor, video analysis and data acquisition were obtained with a video-tracking system (Shanghai Jiliang, China) to analyze total distance, mean velocity and time in the central area.

#### Elevated plus maze

The elevated plus maze (EPM) was conducted using the apparatus (DigBehv-EPMG, Shanghai Jiliang, China) comprised of two open arms (25 cm × 8 cm × 0.5 cm) and two closed arms (25 cm × 8 cm × 12 cm) that extended from a common central platform (8 cm × 8 cm). The apparatus is elevated to a height of 50 cm above the floor. For each test, individual animal was placed in the center square, facing an open arm, and allowed to move freely for 5 min. Mice were videotaped using a camera fixed above the maze and analyzed with a video-tracking system. The entrance is defined as all four paws placed inside an arm. The number of entrance and time spent in each arm were recorded.

#### Forced swim test (FST)

Mice were placed in clear glass cylinder (height: 30 cm, diameter: 16 cm) containing approximately15 cm of water (24 ± 1 °C), so that they could neither escape nor touch the bottom. The apparatus was portioned so that animals were unable to observe animals in the neighboring cylinders. Mice were forced to swim for 6 min. The animals were habituated for the first 1 min and the next 5 min behavior was monitored and scored for immobility time.

### Measurement of oxidative stress markers

Snap-frozen hippocampus was homogenized by ultrasonic wave. An aliquot of the homogenate was used for the assay of malondialdehyde (MDA) formation and superoxide dismutase (SOD) activity. MDA levels and SOD activity were determined using the Lipid Peroxidation MDA Assay Kit and SOD Assay Kit (Nanjing Jiancheng Bioengineering Institute, China), respectively, following the manufacturer’s instructions.

### Histology and immunofluorescence

Male mice were perfused with PBS followed by 4% PFA. Brains were collected and post-fixed in 4% PFA overnight at 4 °C. Tissue samples were embedded in paraffin, cut to a thickness of 4 μm, and stained with hematoxylin and eosin (H&E). For immunostaining analysis, paraffin-embedded tissue cross sections were dewaxed in xylol, rehydrated, antigen retrieval, and then washed with PBS, blocked with 1% BSA (Roche, Switzerland) in PBS for 2 h at room temperature. The sections were incubated at 4 °C overnight with primary antibodies against IBA-1 (1:100, ab178847, Abcam),IL-1β (1:100, ab9722),Nrf2 (1:100, ab62352, Abcam) or 8-hydroxy-2′-deoxyguanosine (8-OHdG,1:200, ab48508, Abcam). This procedure was followed by incubation with FITC/CY3-labeled IgG antibodies (1:1000, Beyotime Biotechnology) for 120 min. DAPI (Thermofisher) was used as nuclear stain. Immunofluorescent images were taken with an inverted fluorescence microscope (IX53, Olympus, Japan).

### Real-time PCR analysis

Total RNA was extracted from the hippocampus using Trizol reagent (Invitrogen, USA) following the manufacturer’s instructions. RNA concentration was determined for quantity and integrity using the spectrophotometry (Jingke, China). cDNA was produced using Revert Aid First Strand cDNA Synthesis Kit (Takara Bio, Japan). Quantitative PCR was performed on Bio-radCx96 Detection System (Bio-rad, USA) using SYBR green PCR kit (Takara Bio, Japan) and gene-specific primers. An amount of 5 ng cDNA sample was used with 40 cycles of amplification. Each cDNA was tested in triplicate. Relative quantitation for PCR product was normalized to β-actin as an internal standard. The sequences of gene specific primers are listed in Table 1.

**Table 1:** Primers used in real-time PCR analyses

Target gene		Primers sequences	Size (bp)
β-Actin	Forward	5′-GGGAAATCGTGCGTGAC-3′	176
	Reverse	5′-AGGCTGGAAAAGAGCCT-3′	
IL-1β	Forward	5′-ATGTGCTGCTGCGAGATTTGA-3′	136
	Reverse	5′-TGCCACCTTTTGACAGTGATG-3′	
IL-6	Forward	5′-TTGGGAGTGGTATCCTCTGTGAA-3′	88
	Reverse	5′-CCTTCTTGGGACTGATGCTGG-3′	
TNF-α	Forward	5′-CACCCCGAAGTTCAGTAGACA-3′	184
	Reverse	5′-TCCCTCCAGAAAAGACACCAT-3’	
Bax	Forward	5′-AGGATGCGTCCACCAAGA-3’	194
	Reverse	5′-AAAGTAGAAGAGGGCAACCAC-3’	
Bcl-2	Forward	5′-CGGGAGAACAGGGTATGA-3’	149
	Reverse	5′-CAGGCTGGAAGGAGAAGAT-3’	
NLRP3	Forward	5′-ATGTTGCCTGTTCTTCCA-3’	130
	Reverse	5′-GGGTCTCCCAGAGTATTG-3’	
ASC	Forward	5′-AGCAAGAGTAAAAGGTGACCG-3’	103
	Reverse	5′-TTGAGTTCATCCCCTGACAAG-3’	
Nrf2	Forward	5′-CAGTGCTCCTATGCGTGAA-3’	109
	Reverse	5′-GCGGCTTGAATGTTTGTC-3’	
HO-1	Forward	5′-ACAGAGGAACACAAAGACCAG-3’	136
	Reverse	5′-GTGTCTGGGATGAGCTAGTG-3’	
NQO1	Forward	5′-TACGATCCTCCCTCAACA-3’	108
	Reverse	5′-TACAGCAGCCTCCTTCAT-3’	

### Western blot analysis

Tissue samples were homogenized in ice-cold RIPA buffer supplemented with a protease inhibitor cocktail. The concentration was determined using a BCA Protein Assay Kit (Beyotime Biotechnology, China). Samples were then subjected to western blotting analysis. Equal amounts of protein (50 μg) from the hippocampus were separated by SDS–polyacrylamide gel electrophoresis using 4~20% tris-glycine gel. After transfer of proteins to PDVF membranes, blots were blocked in 5% non-fat milk for 1 h at room temperature and incubated with the respective primary antibody at 4 °C overnight. Primary antibodies were as follows: NLRP3 (Abcam, ab214185;1:500), ASC (Abcam,ab175449; 1:1000), caspase-1 (Abcam, ab1872; 1:1000), Nrf2 (Abcam, ab62352, 1:1000), HO-1 (Proteintech,10,701–1-AP, 1:1000), NQO-1 (Abcam, ab28947, 1:1000),TXNIP (Abcam, ab188865, 1:1000), Trx (Abcam, ab26320, 1:500), and β-actin (Proteintech, 20,536–1-AP; 1:4000). Following incubation with the appropriate HRP-conjugated secondary antibodies, the immunological complexes were visualized with enhanced chemiluminescence detection kit (abs920, Absin Bioscience Inc.).

### Statistical analysis

All the results are expressed as the mean ± SEM. Statistical analyses of the data were performed using SPSS. The number of animals varied per experiment and is noted in the corresponding figure legend. Differences between groups were determined by one-way or two-way analysis of variance (ANOVA) test, followed by Tukey’s test for post hoc comparisons. The prior level of significance was established at *p* < 0.05.

## Results

### PCMS promotes recovery from LPS-induced behavioral deficits

Mice were treated with either a low (200 μg/kg) or a moderate (500 μg/kg) dose of LPS, to examine the dose–response relationship of peripheral LPS injection on behavioral changes in stress naïve and PCMS mice. Our results showed that under a lower dose LPS challenge, early stage sickness behavior as indicated by decreased locomotor distance and mean velocity was partially normalized in PCMS mice when compared with stress naïve mice. At 24 h time point, stress-naïve mice receiving LPS displayed comparable locomotor activity with that of saline injected mice indicating a self-recovery of sickness symptoms (Additional file 1: Figure S1). On the contrast, at the dose of 500 μg/kg and 4 h post treatment, locomotor activity in mice subjected to LPS was much lower than that displayed by saline-injected mice either from stress naïve group or PCMS group (Fig. 1b and c). Then, OFT, FST, and EPM tests were used to evaluate depressive-like and anxiety-like behavioral alterations 24 h post LPS treatment. Stress naïve mice showed significantly larger locomotor activity drops (Fig. 1c) and lower mean velocity of locomotor activity (Fig. 1d) compared with PCMS mice 24 h post LPS treatment. Mice receiving LPS from stress naïve but not PCMS group displayed a significant reduction in time spent in the central area compare with that of saline injected mice (Fig. 1e). Furthermore, a longer immobility time in the FST (Fig. 1f) and larger reduction in time spent in the open arms in the EPM test (Fig. 1g) were observed in stress naïve mice when compared with PCMS mice following LPS treatment. These results indicate that PCMS promotes recovery from LPS-induced behavioral deficits.

### PCMS mitigates LPS-induced neuroinflammation

**Fig. 2:**
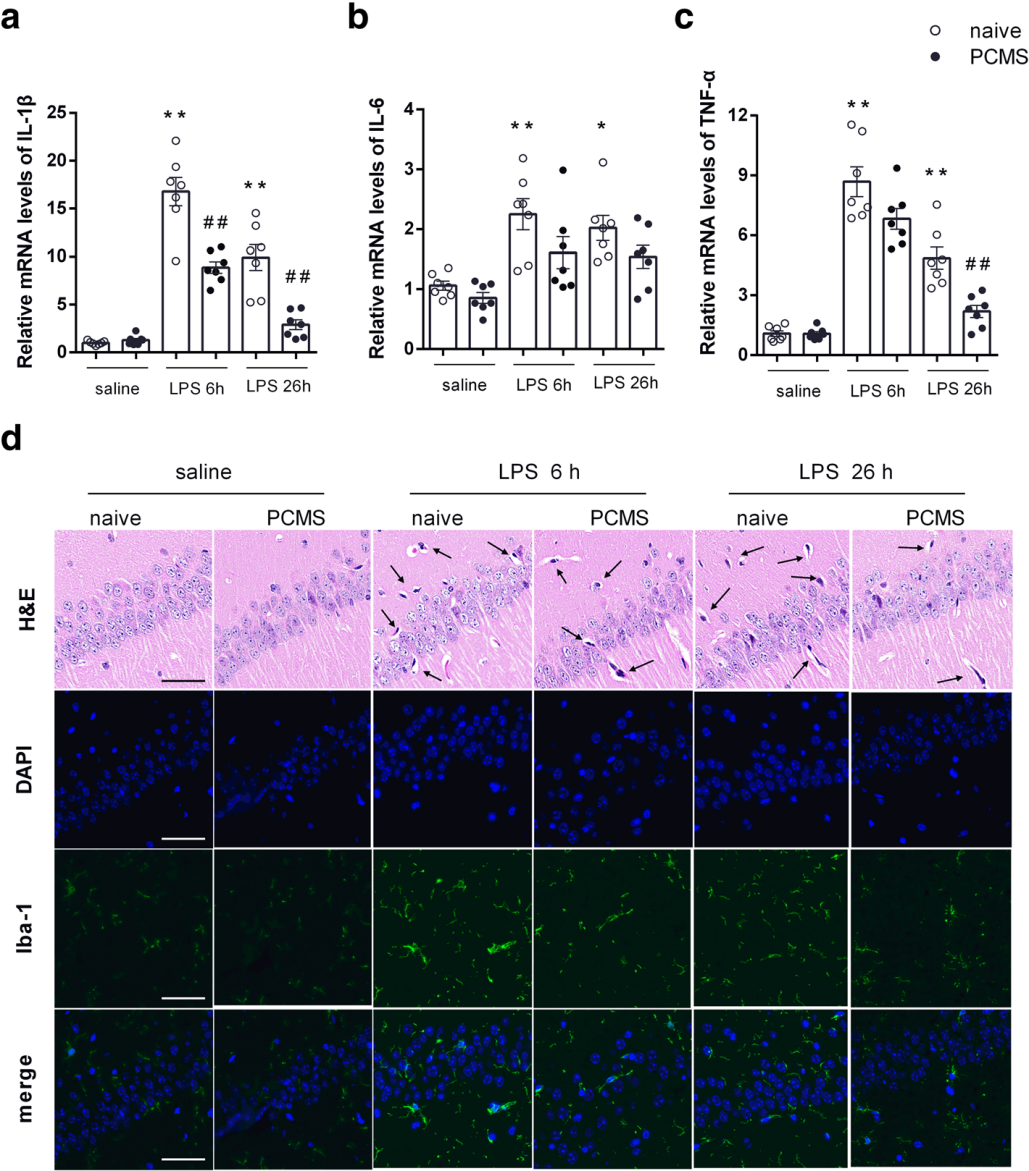
PCMS mitigates LPS-induced neuroinflammation. Relative mRNA expression of the proinflammatory cytokines, IL-1β (**a**), IL-6 (**b**), and TNF-α (**c**) in the hippocampus of mice brain 6 h and 26 h after saline or LPS (500 μg/kg) treatment. Histological changes and microglial activation (Iba-1 immunofluorescence) in the hippocampal CA1 region (**d**). Scale bar = 50 μm. Data are means ± SEM (*n* = 7). *p < 0.05, **p < 0.01 compared with the saline-treated naive group. ^##^*p* < 0.01 compared with the corresponding LPS group

Hippocampus is a brain region critical for memory and the regulation of emotion. Notably, hippocampus contains the highest microglia density and is highly sensitive to stress [23]. We then examined whether the neuroinflammatory response would differ in hippocampus of mice with or without PCMS following LPS administration. As shown in Fig. 2a–c, mRNA levels of IL-1β, IL-6, and TNF-α in the hippocampus were significantly enhanced in stress naïve mice6 h post LPS treatment compare with saline treated mice. Meanwhile, PCMS mice showed comparable gene expression levels of proinflammatory cytokines with those in stress naïve mice except a reduced IL-1β mRNA level. At 26 h post LPS treatment, stress naïve mice subjected to LPS showed a noteworthy increase in the mRNA levels of IL-1β, IL-6, and TNF-α compared with saline treated mice, whereas PCMS mice subjected to LPS displayed reduced upregulation of IL-1βand TNF-α when compare with stress naïve mice. The neuronal histopathological changes in the hippocampus were observed by H&E staining. As shown in Fig. 2d, the neurons in the hippocampus of stress naïve mice showed nuclear condensation and acidophilic degeneration after LPS treatment; these neuronal injuries were alleviated in PCMS mice. Immunofluorescence for ionized calcium-binding adapter molecular 1 (IBA-1) was used for the analysis of microglia activation. In accordance with increased expression of proinflammatory cytokines, enhanced IBA-1 expression in stress naïve mice was observed at 6 h and 26 h post LPS administration, whereas LPS induced microglia activation was attenuated in PCMS mice.

### PCMS abrogates LPS-induced oxidative stress

**Fig. 3:**
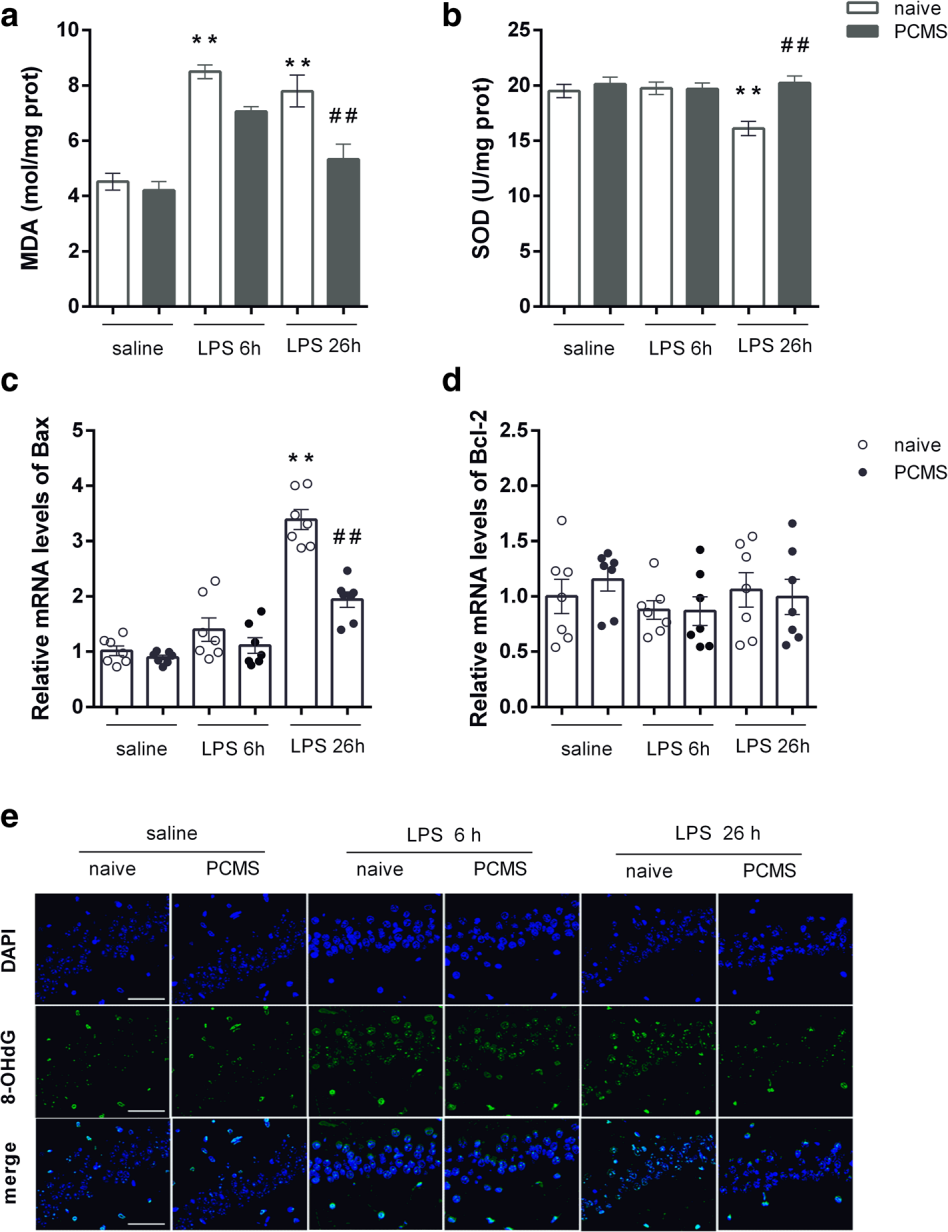
PCMS abrogates LPS-induced oxidative stress. Brain MDA level (**a**) and SOD activities (**b**). Relative mRNA expression of apoptotic markers Bax (**c**) and Bcl-2 (**d**) in hippocampus. The immunostaining images of 8-OHdG in the hippocampal CA1 region (**e**). Scale bar = 50 μm. Data are means ± SEM (*n* = 6–7). *p < 0.05, **p < 0.01 compared with the saline-treated naive group. ^#^*p* < 0.05, ^##^*p* < 0.01 compared with the corresponding LPS group

Parallel to the increased expression of proinflammatory cytokines, MDA production in hippocampus of stress naïve mice was significantly increased at 6 h and 26 h post LPS administration (Fig. 3a), and SOD activities were reduced 26 h post LPS treatment (Fig. 3b). 8-OHdG is a sensitive biomarker of oxidative DNA damage, whose expression in hippocampus of stress naïve mice was also significantly higher following LPS administration. PCMS was able to ameliorate the LPS-induced oxidative stress in the hippocampus, with decreased MDA level (Fig. 3a), enhanced SOD activities (Fig. 3b) and reduced 8-OHdG (Fig. 3e) expression 26 h post LPS administration when compared with that in stress naïve mice. In addition, gene expression of pro-apoptotic Bax was largely increased in the hippocampus of stress naïve mice 26 h post LPS administration (Fig. 3c), and reduced in PCMS mice compared with stress naïve mice. No significant gene expression changes of Bcl-2 were observed from either stress naïve or PCMS mice 26 h post LPS administration (Fig. 3d).

### PCMS ameliorates LPS-induced NLRP3 overexpression

**Fig. 4:**
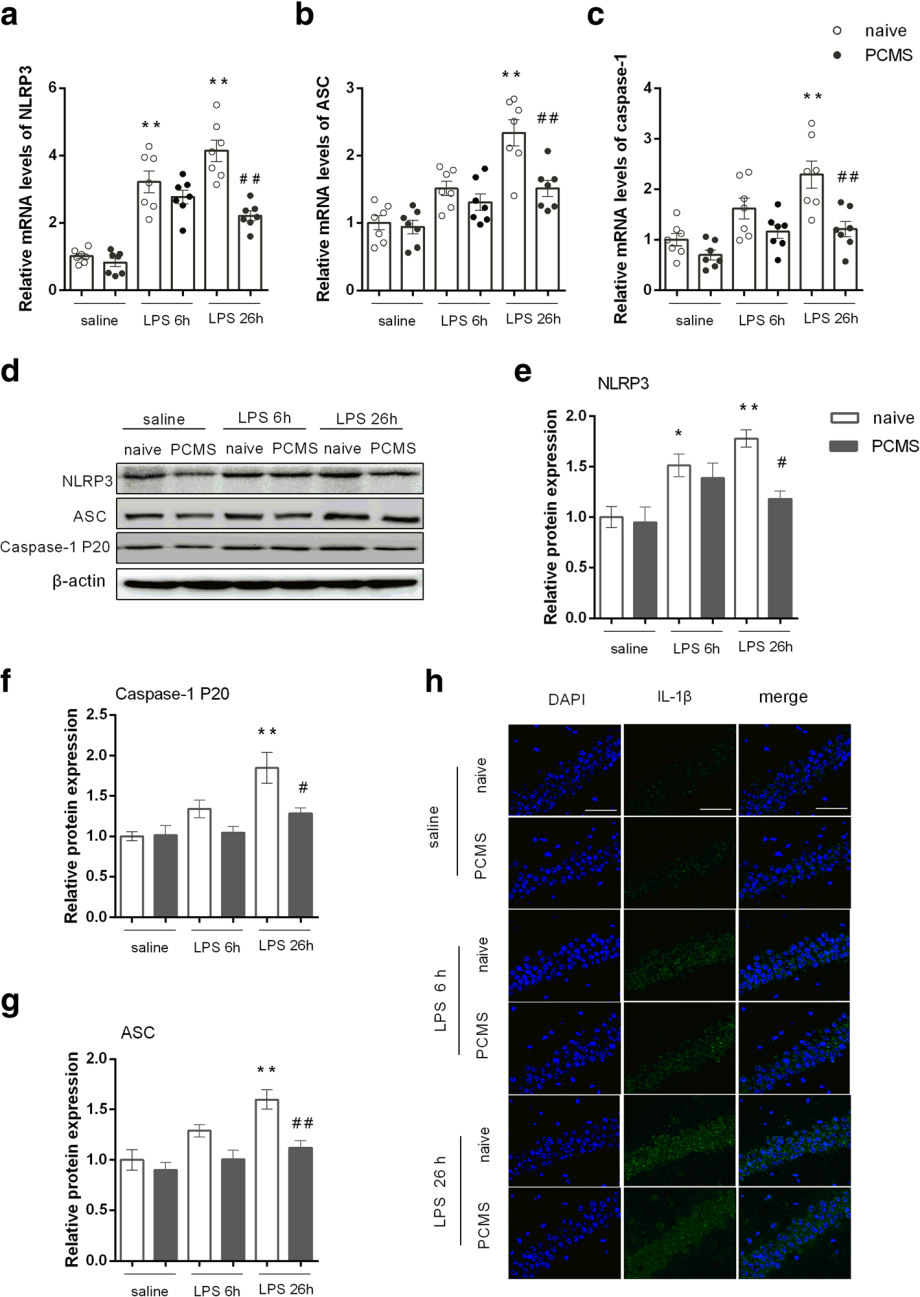
PCMS ameliorates LPS-induced NLRP3 overexpression. Relative mRNA expression of NLRP3 (**a**), ASC (**b**), and caspase-1 (**c**). Representative blots of NLRP3 and inflammasome components (ASC and caspase-1 P20) and mature IL-1β (**d**). Statistical graphs of relative protein expression of NLRP3 (**e**), ASC (**f**), and caspase-1 P20 (**g**). The immunostaining images of IL-1β in the hippocampal CA1 region (**h**). Scale bar = 50 μm. Data are means ± SEM (*n* = 6). *p < 0.05, **p < 0.01 compared with the saline-treated naive group. ^#^*p* < 0.05, ^##^*p* < 0.01 compared with the corresponding LPS group

Then, we analyzed the expression of NLRP3 inflammasome components. We found that LPS significantly increased NLRP3 mRNA (Fig. 4a) and protein levels (Fig. 4e) in the hippocampus of stress naïve mice either 6 h or 26 h post LPS administration. In parallel, LPS also induced expression of ASC (Fig. 4b and f) and significant activation of caspase-1 (cleaved caspase-1 P20) (Fig. 4g) 26 h post administration. IL-1β was also significantly increased in stress naïve mice following LPS injection (Fig. 4h). On the contrary, PCMS mice showed partially inhibited NLRP3 inflammasome activation as evident by significant decrease in the protein levels of NLRP3 inflammasome components and attenuated IL-1β expression when compared with stress naïve mice 26 h post LPS administration. These results suggest that PCMS could ameliorate hippocampal NLRP3 inflammasome activation and interleukin-1 maturation in mice subjected to LPS challenge.

### PCMS alleviates LPS-induced impairment of Nrf2-TXNIP-Trx signaling

**Fig. 5:**
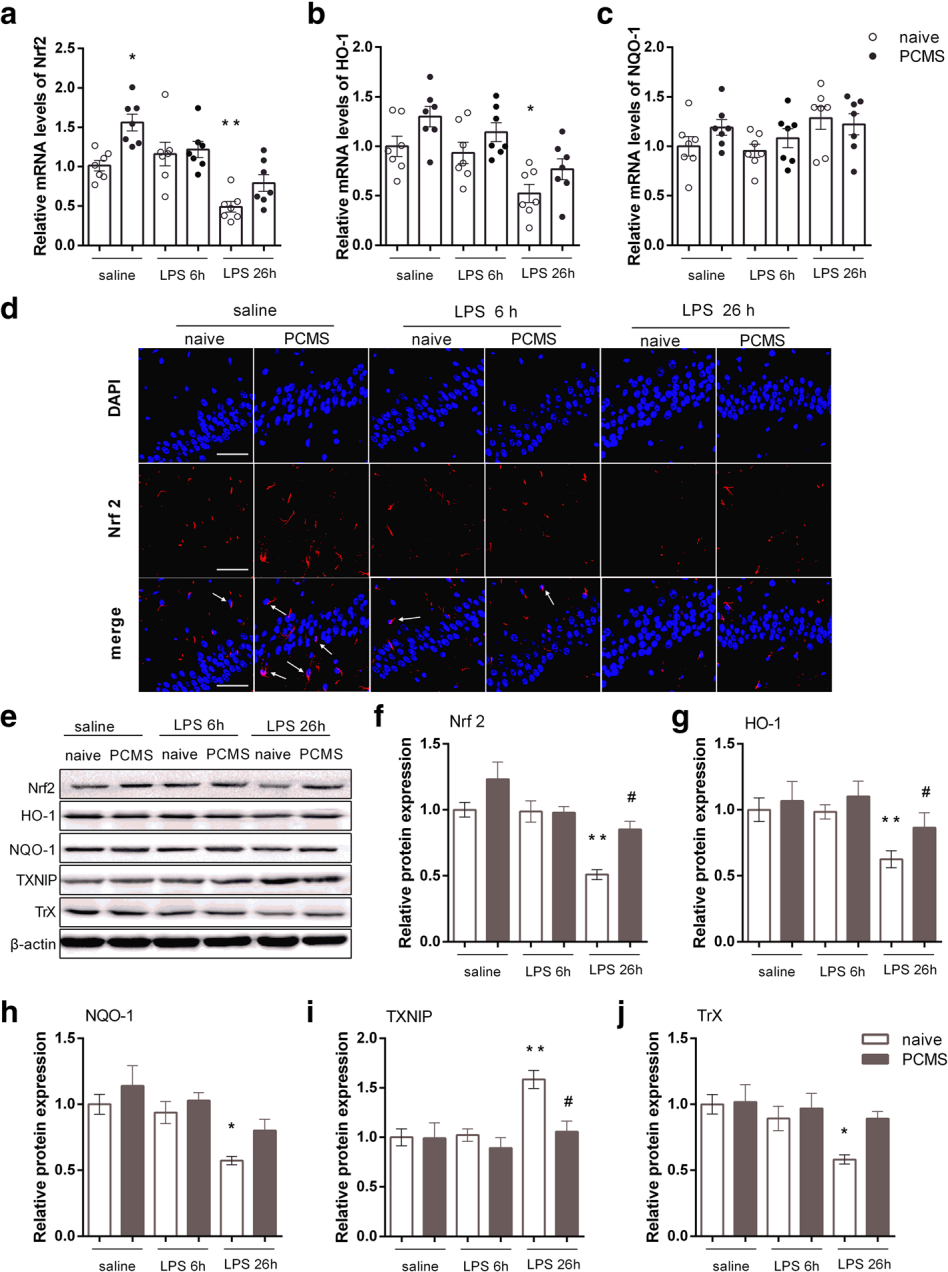
PCMS alleviates LPS-induced impairment of Nrf2-TXNIP-Trx signaling. Relative mRNA expression of Nrf2 (**a**) and Nrf2-downstream antioxidant genes HO-1 (**b**), and NQO-1 (**c**). Immunofluorescence staining images of Nrf2 in the hippocampal CA1 region (**d**). Representative blots (**e**) and statistical graphs of relative protein expression of Nrf2 (**f**), HO-1 (**g**), NQO-1 (**h**), TXNIP (**i**), and Trx (**j**). Scale bar = 50 μm. Data are means ± SEM (*n* = 6). *p < 0.05, **p < 0.01 compared with the saline-treated naive group. ^#^*p* < 0.05, ^##^*p* < 0.01 comparedwith the corresponding LPS group

We further assessed the hippocampal expression of Nrf2 and downstream antioxidant proteins HO-1 and NQO-1given the critical role of Nrf2 system in the regulation oxidative stress and inflammasome activation. As shown in Fig. 5a, f, mRNA and protein levels of Nrf2 in stress naïve mice were inhibited 26 h post LPS administration compared with saline-treated mice. In line with the downregulated Nrf2, the levels of Nrf2-dependent proteins HO-1 (Fig. 5g) and NQO-1(Fig. 5h) were apparently lower in the hippocampus of the LPS-treated stress naïve mice than those in the saline-treated mice. Though significant difference in protein levels of Nrf2 was not observed, PCMS mice showed increased gene expression of Nrf2 compared with stress naïve mice (Fig. 5a). Immunofluorescence staining (Fig. 5d) reveals that the expression of Nrf2 protein is more concentrated in the nucleus in PCMS mice as compared with stress naïve mice, indicting an increased translocation of Nrf2 from the cytosol to the nucleus. Furthermore, a significantly upregulated trend of Nrf2 and HO-1 was found in PCMS mice when compared with stress naïve mice 26 h post LPS challenge (Fig. 5f and g). We then examined the levels of Trx1/TXNIP complex by Western blot. As shown in Fig. 5i and j, protein levels of TXNIP were significantly increased and Trx1 was attenuated in hippocampus of stress naïve mice 24 h post LPS challenge. On the contrast, the expression of TXNIP was significantly decreased, and a non-significant upregulated trend of Trx1 was observed in PCMS mice when compared with stress naïve mice 26 h post LPS challenge. These results indicate that PCMS alleviates LPS-induced impairment of Nrf2-TXNIP-Trx signaling.

## Discussion

The current study shows that PCMS, characterized by predictability, mild intensity, and appropriate duration of stress exposure, increases resistance and aids recovery from LPS-induced abnormal behavioral changes. An inverted-U-shaped dose response relationship is proposed to explain the consequences of stress on brain health [21]. The stress reaction can be severely damaging when the stress exceeded the individual’s endurance, inducing aberrant adaptation and long-term pathological responses. Consistent with our findings, previous studies using similar stress paradigms enhanced cognitive function, stimulated adult hippocampal neurogenesis and relieved depressive and anxiety-like behavior [22, 23]. Similarly, some other controllable and mild stressors such as intermittent social separations [24], exercise [25] and environmental enrichment [26] help develop adaptive stress responses and exert anti-depressive and anxiolytic effects in various animal models. These studies, along with our findings, evoke the interesting possibility that a history of stress exposure may be beneficial for brain function and influence the future stress reactivity, providing a plausible explanation for individual differences in stress susceptibility.

MDD is a multifaceted disease. The past decade has witnessed a breakthrough in our understanding of the mechanistic basis for MDD and revealed neuroinflammation as an important mediator of MDD [8]. A previous study found that LPS-stressed mice showed more remarkable behavioral changes and robust expression and release of IL-1β, IL-6 and TNF-α in brain compared with four-week UCMS-stressed mice [27]. The present study uses peripheral immune challenge with LPS to model depressive state. In line with previous findings, brain inflammation was observed after peripheral LPS injection, as confirmed by microglial activation and upregulated expression of proinflammatory cytokines. We further showed that PCMS-alleviated behavioral changes were accompanied by the attenuation of neuroinflammatory responses, consistent with our previous studies that link neuroimmune modulating features of omega-3 polyunsaturated fatty acids with their antidepressant actions [28, 29]. Our observation is also consistent with previous evidence, showing that two phytochemicals derived from polyphenol were effective in promoting resilience against chronic stress-induced depression-like phenotypes by reversing stress-mediated brain synaptic maladaptation through modulation of proinflammatory IL-6 generations [30]. Significant roles of inflammatory cytokines in mediating resilience and susceptibility to depression are also supported by recent findings that social defeat stress-induced neurovascular pathology and increased blood-brain barrier permeability, thereby facilitating peripheral cytokine infiltration into brain subsequent expression of depression-like behavior [31]. Chronic stressors can activate microglial cells and inflammatory responses, leading to increased cytokines and contributing to structural and functional alterations of neurons and behavioral abnormalities. The question on the different influences of PCMS and intense stress on the neuroinflammatory responses remains. In a previous work, the transfer of stress-modified lymphocytes into lymphopenic naive Rag^2−/−^mice exerted antidepressant effects, indicating an immunological memory within the adaptive immune system to confer stress resilience when it has been primed by stress [32].On the contrary, microglia, as the brain resident macrophage, has strong plasticity and diversified functional phenotypes that range from proinflammatory M1(classical activated) phenotypes to immunosuppressive M2 (alternative activated) phenotypes. The activation profileof microglia may transform from M1 type to M2 type, depending on the stimulus encountered, thereby producing either cytotoxic or neuroprotective effects [33, 34]. In summary, these data raise the possibility that stress could prime the immune system to confer stress resiliency to its host.

The activation of NLRP3 inflammasome has shown to contribute to the depression-like behavior in animals caused by either stress or LPS [28, 35]. Following NLRP3 inflammasome activation, cleaved caspase-1 will cleave pro-IL-1β into its biological active form, mature IL-1β, which is then released into the extracellular environment. The present data show that PCMS attenuated LPS-induced NLRP3 inflammasome activation with inhibited expression of the key components (NLRP3, ASC, caspase-1 P20) and mature IL-1β. Our findings are in agreement with earlier work, showing that genetic knock-out or inhibition of NLRP3 increases the resilience to stress and LPS-induced inflammatory responses and behavioral abnormities [35–37]. In addition to the alleviated neuroinflammation, markers reflecting the status of oxidative stress, such as MDA and 8-OHdG, were inhibited in PCMS mice following LPS administration. Moreover, we found that PCMS enhanced expression of Nrf2 and the targeting cytoprotective enzyme HO1. Nrf2, which plays a central role in cellular defense against oxidative insults, has been shown to be involved in the restoration of NLRP3 overactivation in various studies, and proposed as a potential target for the treatment of neuropsychiatric disorders [17, 38]. Nrf2 gene deletion was sufficient to induce depressive-like behavior in mice, whereas anti-inflammatory treatment with rofecoxib or induction of Nrf2 by sulforaphane could prevent LPS-induced behavioral abnormalities [39]. In addition, a recent study on a animal model of intense stress (social defeat) shows that vulnerability to depression-like phenotype resulted from a persistent state of oxidative stress, and activating Nrf2 translocation could restore redox homeostasis and abolish the vulnerability to depression [18]. Furthermore, increased levels of inflammation and oxidative stress marker in MDD patients were associated with worse antidepressant treatment response [40]. These data provide consistency to our hypothesis that upregulated Nrf2 signaling in the brain may contribute to neuroprotection of PCMS via enhanced antioxidative defense and anti-inflammatory effects.

In our study, PCMS suppressed LPS-induced expression of TXNIP, which is consistent with the reported indispensable role of TXNIP in activation of the NLRP3 inflammasome [19]. Furthermore, previous studies demonstrated that Nrf2 negatively regulates the NLRP3 inflammasome in a Trx1/TXNIP dependent manner [16, 20]. Gene array revealed that Nrf2 controls the gene expression of TXNIP, acting as a key gatekeeper of TXNIP transcription to keep TXNIP expression at a low level [41]. Thus,Nrf2/ Trx1/TXNIP signaling link oxidative stress to neuroinflammation given the central roles in defense against oxidative stress, thereby making our findings mechanistically more comprehensible. In line with our findings, a recent study has demonstrated that ablation of uncoupling protein 2 (UCP2), which has been well characterized to control the production of reactive oxygen species, significantly enhanced the activation of NLRP3 inflammasome in the hippocampus and aggravated chronic stress induced depressive-like behavior in mice. Further examinations revealed that UCP2 deficiency promoted TXNIP-NLRP3 association and contributed to the enhancement of NLRP3 activation in UCP2 knockout astrocytes [42]. Another research showed that antidiabetic drug glyburide abrogated 12-week CUMS induced depressive-like behavior and insulin resistance through inhibiting hippocampal NLRP3 inflammasome activation via downregulating TXNIP [43]. In summary, our results may also provide a novel strategy for the prevention of MDD and comorbid diseases commonly due to chronic cytokine mediated inflammatory responses. Brain is particularly susceptible to perturbations of homeostasis. Neuroinflammation along with oxidative stress could constitute a vicious cycle, thereby contributing to aggravated neural damage. Our previous studies extensively described the intensive interaction between inflammation and many of the pathophysiological domains that characterize depression in the context of neuroinflammation, including neurotransmission, neural plasticity, and neurotrophic network [11, 44, 45]. The brain’s response to stress can also define the line between health-promoting adaptation and long-term pathology that predispose individuals to MDD after chronic stress. Enhanced Nrf2-TXNIP-Trx system function may provide a strong adaptive capacity to buffer stress-induced inflammatory damage and disturbances of cellular redox homeostasis, thereby building the resilience to negative consequences of stress.

In summary, our data showed that specific predictable mild stress exposure may help develop adaptive capacity and become more resilient to negative effects to future stressors. We showed, in a straightforward fashion, using an inflammation-induced model of depression that PCMS confers antidepressant effects via alleviated neuroinflammation and oxidative stress. NLRP3 inflammasome and Nrf2-TXNIP-Trx signaling, known as stress response pathways, may help the individuals to buffer future stress responses following PCMS. This investigation was designed as a proof of concept study, mainly focused on the phenomena but with limited underlying mechanisms. Further studies will be required to better understand the complicated connection among stress response system, neuroendocrine network, and MDD susceptibility to alleviate the burden of mental illness.

## Additional file

Additional file 1: **Figure S1.** PCMS alleviates LPS-induced behavioral changes. Sample traces of locomotor activity in the open field test (OFT) of naïve and PCMS mice 4 h and 24 h after saline or LPS (200 μg/kg) treatment (a). Total distance traveled (b), mean velocity (c) of locomotor activity and time spent in the center area (d) in the OFT. Immobile time in the forced swim test 24 h after saline or LPS (e). Time spent in the open arms (f) and entrance to open arms (g) in the elevated plus maze test. (*n* = 7–9). *p < 0.05, **p < 0.01 compared with the saline-treated naive group. ^#^*p* < 0.05, ^##^*p* < 0.01 compared with the corresponding LPS group. (DOCX 394 kb)

## Abbreviations

ASC: Apoptosis-associated speck-like protein containing a CARD
CNS: Central nervous system
H&E: Hematoxylin and eosin
HO: Heme oxygenase
HPA: Hypothalamic–pituitary–adrenal
IL: Interleukin
LPS: Lipopolysaccharide
NLRP3: Nod-like receptors family pyrin domain containing 3
Nrf2: Nuclear factor erythroid 2-related factor 2
TNF: Tumor necrosis factor
Trx: Thioredoxin
TXNIP: Thioredoxin interacting protein
