# Supplementary material for: Predictable chronic mild stress promotes recovery from LPS-induced depression
Source: Mol Brain. 2019 May 3;12:42. doi: 10.1186/s13041-019-0463-2 (PMC6500057; doi:10.1186/s13041-019-0463-2)
Supplement: Supplementary file 1 — Figure S1. PCMS alleviates LPS-induced behavioral changes. Sample traces of locomotor activity in the open field test (OFT) of naïve and PCMS mice 4 h and 24 h after saline or LPS (200 μg/kg) treatment (a). Total distance traveled (b), mean velocity (c) of locomotor activity and time spent in the center area (d) in the OFT. Immobile time in the forced swim test 24 h after saline or LPS (e). Time spent in the open arms (f) and entrance to open arms (g) in the elevated plus maze test. (n = 7–9). *p < 0.05, **p < 0.01 compared with the saline-treated naive group. #p < 0.05, ##p < 0.01 compared with the corresponding LPS group. (DOCX 394 kb) [file 13041_2019_463_MOESM1_ESM.docx]

**

**

**Additional file 1 Figure S1.** **PCMS alleviates LPS-induced behavioral changes.** Sample traces of locomotor activity in the open field test (OFT) of naïve and PCMS mice 4 h and 24 h after saline or LPS (200 μg/kg) treatment **(a)**. Total distance traveled **(b)**, mean velocity **(c)** of locomotor activity and time spent in the center area **(d)** in the OFT. Immobile time in the forced swim test 24 h after saline or LPS **(e)**. Time spent in the open arms **(f)** and entrance to open arms **(g)** in the elevated plus maze test. (n = 7-9). *p < 0.05, **p < 0.01 compared with the saline-treated naive group. ^#^p < 0.05, ^##^p < 0.01 compared with the corresponding LPS group
